# Energy requirements of female sheep at increasing ages and fed at different concentrate levels

**DOI:** 10.1007/s11250-026-04903-3

**Published:** 2026-03-02

**Authors:** Antonio de Sousa Brito Neto, Izabelle Auxiliadora Molina de Almeida Teixeira, Marcos Inácio Marcondes, Caio Julio Lima Herbster, Evandra da Silva Justino, Leilson Rocha Bezerra, Stefanie Alvarenga Santos, Ronaldo Lopes Oliveira, Elzania Sales Pereira

**Affiliations:** 1 Department of Animal Science, Federal University of Viçosa, Viçosa, MG 36571-000 Brazil; 2 Department of Animal, Veterinary and Food Sciences, University of Idaho, 11 ID-1827, Twin Falls, 83303 USA; 3 William H. Miner Agricultural Research Institute, Chazy, NY 12921 USA; 4 Department of Animal Science, Federal University of Ceara, Fortaleza, Ceara 60356000 Brazil; 5 Department of Animal Science, Federal University of Campina Grande, Patos, Paraíba 58708110 Brazil; 6 School of Veterinary Medicine and Animal Science, Federal University of Bahia, Salvador, Bahia 40170110 Brazil

**Keywords:** Comparative slaughter, Growth, Hair sheep, Small ruminants

## Abstract

This study aimed to estimate the energy requirements of Santa Ines females. Forty Santa Ines females with an average initial body weight (BW) of 16.27 ± 1.44 kg and an age of 150 ± 10 days were used. Four animals were slaughtered on the first day of the experimental period to serve as a baseline group. The remaining 36 females were assigned to a completely randomized design in a factorial arrangement with different concentrate levels [50% concentrate *ad libitum*(CL1), 25% concentrate *ad libitum*, and 25% concentrate with restricted intake at 1.9% of body weight (CL2R)] and slaughter ages [200 days (A1), 250 days (A2) and 330 days (A3)]. The net energy requirements for maintenance (NE_m_) were estimated through regression analysis of heat production (HP) on metabolizable energy intake (MEI). The net energy requirements for gain (NE_g_) were estimated by regressing retained energy (RE) on empty body weight gain (EBWG). Energy concentration of gain increased with age (*P* = 0.053), and was higher (*P* = 0.021) in females fed a 50% concentrate diet. The NE_m_ was estimated at 0.077 Mcal/kg^0.75^EBW/day. The model generated for NE_g_was 0.4489 × EBW^0.75^ × EBWG^1.0284^. The energy requirements of Santa Ines females remain constant up to 330 days of age.

## Introduction

Livestock production in the tropics is of great importance on the world stage, due to the large number of animals present in these regions as well as the number of people who benefit from this activity (Oosting et al. 2014). These regions are characterized by high temperatures, often associated with seasonality in food supply (Regadas Filho et al. 2013). In this context, the production systems present in these regions require genetic groups that are able to withstand adverse conditions imposed by the environment (Costa et al. 2013). Hair sheep and their crosses stand out due to their resistance and ability to adapt to tropical and semi-arid climates (Pereira et al. 2018), being mainly used in meat production (Pereira et al. 2014).

Nutrition is the item with the highest operational cost in production systems, which highlights the importance of diet formulations being based on precise and accurate models for predicting nutritional requirements, so that productive and economic efficiency can be achieved (Valadares Filho et al. 2009). However, diet formulations are still based on recommendations from International Committees developed under different environmental conditions and with the use of genotypes distinct from those found in tropical and semi-arid regions (AFRC 1993; NRC 2007; CSIRO 2007). Thus, the recommendations established by these Committees may not be adequate for the genotypes present in the tropics (Oliveira et al. 2018; Herbster et al. 2024). Therefore, determining the nutritional requirements of hair sheep is essential for developing more precise and effective feeding strategies, aiming to increase productivity and profitability in the production system.

The Santa Ines breed is one of the most popular in Brazil and is distributed throughout the country (McManus et al. 2014) due to its adaptability to the tropical climate and its acceptable production potential (Regadas Filho et al. 2013). Due to their greater hardiness and prolificacy, lower reproductive seasonality, and small size when compared to other specialized breeds, Santa Ines sheep have been frequently used for the production of top-quality lambs, whether purebred or crossbred with meat sheep breeds (Ribeiro and González-García 2016).

Additionally, although knowledge of the nutritional requirements of hair sheep has advanced in recent years, studies mainly include intact and castrated males (BR-Caprinos & Ovinos 2024; Herbster et al. 2024). For females, there is little information about their nutritional needs (Rodrigues et al. 2016; Pereira et al. 2018), and no published information on growing females of the Santa Ines breed. Furthermore, studies that seek to estimate nutritional requirements are necessary to expand data sets to support future compilations (Oss et al. 2017), as well as updates and validation of more consistent models.

Metabolic rate per unit of weight is higher in younger animals than in adults. As the animal ages, the curvilinear decrease with age may be partially a result of the relative differences in growth rates of tissues and organs. Brody (1945) summarized data on numerous species that demonstrated that the proportion of liver and intestine weight to live weight decreases with increased weight in maturing animals. In models developed to estimate organ and tissue growth in rams (Jenkins and Leymaster 1993), the liver and empty gastrointestinal tract weight as a proportion of live weight decreases curvilinearly as the animal ages. According to CSIRO 2007, fasting metabolism decreases with age, at about 8% per year in the young animal, with the rate falling to zero at about six years age, by which time fasting metabolism is about 0.84 of the initial value. We hypothesized that although body composition changes, energy requirements for maintenance remain constant in first year of age. This study aimed to evaluate the intake, digestibility, performance, body composition, energy flow, and estimate the energy requirements of Santa Ines females fed different levels of concentrate and at increasing ages.

## Material and methods

### Site

The experiment was conducted in the Animal Nutrition Laboratory of the Department of Animal Science at the Federal University of Ceará, in Fortaleza, Ceará, Brazil (30°43′02′′S, 33°32′35′′W). Throughout the experiment, the average daily minimum and maximum air temperatures were 24.6 °C ±0.82 and 31.2 °C ±1.32, respectively, and the minimum and maximum relative humidity were 71.1% ± 7.58 and 89.1% ± 4.27, respectively.

### Animals and feeding management

Forty Santa Ines female sheep with an average initial body weight (BW) of 16.27 ± 1.44 kg and an age of 150 ± 10 days were used, all sourced from the same commercial herd. Initially, all animals were identified, weighed, dewormed, vaccinated against clostridiosis, supplemented with vitamins, and placed in individual pens equipped with feed and water troughs. After a 20-day adaptation period to the diet and management conditions, four females were slaughtered on the first day of the experimental period to serve as a baseline group.

### Treatments and experimental design

**Fig. 1:**
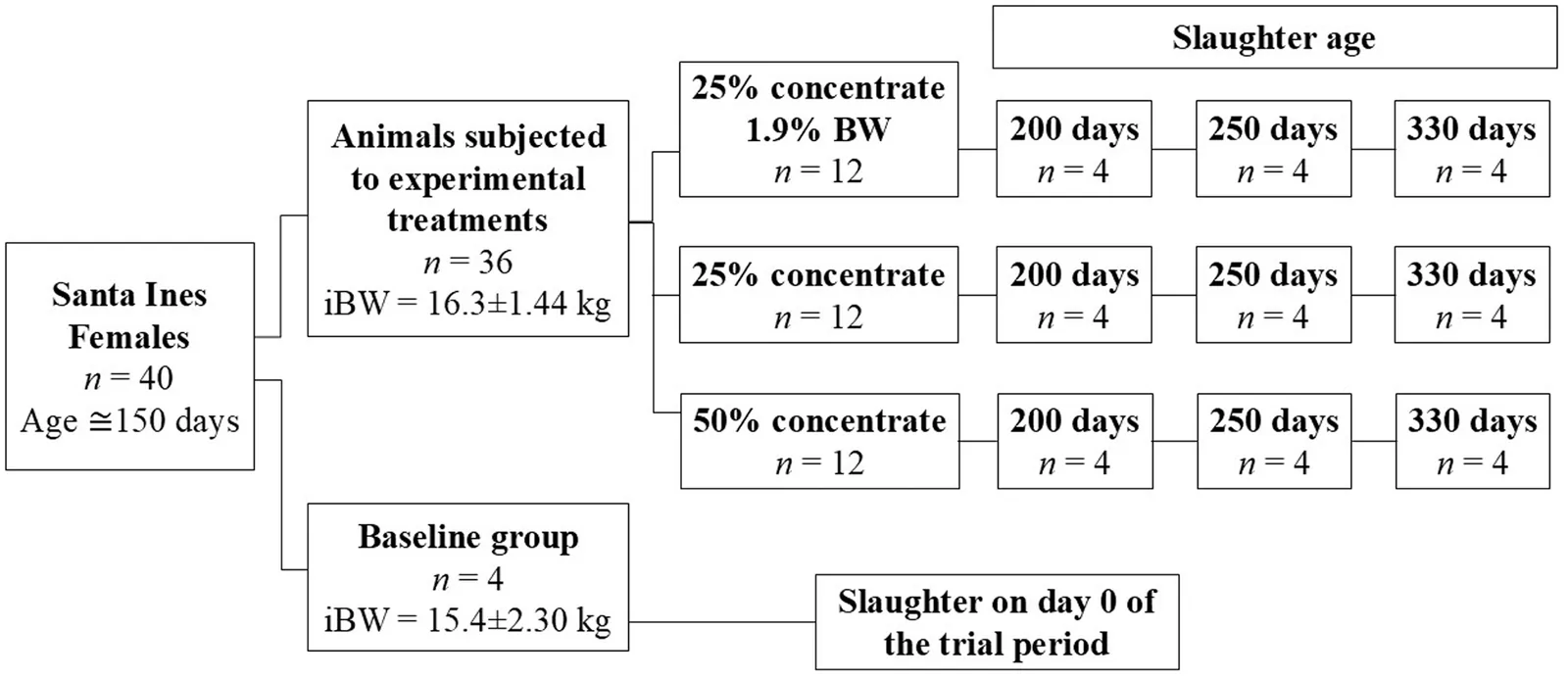
Experimental scheme, experimental diets, and slaughter groups. iBW, initial body weight

The remaining 36 females were assigned to a completely randomized design in a factorial arrangement with different concentrate levels [50% concentrate *ad libitum* (CL1), 25% concentrate *ad libitum*, and 25% concentrate with restricted intake at 1.9% of body weight (CL2R)] and slaughter ages [200 days (A1), 250 days (A2) and 330 days (A3)]. The animals were randomly assigned to experimental diets. The experimental scheme is shown in Fig. 1. Animals fed at a CL2R (maintenance level) were necessary for the assessment of energy flow and estimation of energy requirements. Diets were formulated with 13% crude protein for a daily gain of 150 g (Tables 1 and 2).

**Table 1:** Chemical composition of the ingredients and concentrated rations

Item	DM (g/kg)	CP (g/kg)	EE (g/kg)	MM (g/kg)	OM (g/kg)	**NDF**_**ap**_**(g/kg)**	NFC (g/kg)	GE (Mcal/kg)
Tifton hay	907.89	96.67	23.10	92.10	907.90	695.40	92.70	4.49
Corn ground	879.52	82.95	45.10	12.20	987.80	157.50	702.20	4.66
Soybean meal	900.37	525.66	16.30	74.60	925.40	164.80	218.60	4.90
50% ration	896.58	180.75	42.10	27.80	972.20	162.00	587.30	4.52
25% ration	896.03	246.28	35.60	42.10	957.90	157.20	518.80	4.62

DM = dry matter; CP = crude protein; EE = ether extract; MM = mineral matter; OM = organic matter; NDF < sub icoretag=“sub” isexistfmt=“true” aid=“5ix2s08614v7ay3” ia_version=“0” > ap  = neutral detergent fiber corrected for ash and protein; NFC = non-fibre carbohydrates; GE = gross energy

**Table 2:** Ingredients and chemical composition of the experimental diets

Item	Concentrate levels	
	50(%)	25(%)
Formulation (g/kg)		
Tifton 85 grass hay	500.00	750.00
Ground corn	388.16	155.90
Soybean meal	107.23	91.39
Dicalcium phosphate	2.72	1.59
Limestone	1.96	1.22
Composition (g/kg)		
Dry matter	902.23	904.92
Crude protein	138.71	134.07
Ether extract	32.60	26.23
Mineral matter	59.95	79.60
Organic matter	940.05	920.40
Neutral detergent fiber corrected for ash and protein	428.70	560.85
Non-fiber carbohydrates	340.00	199.23
Energy density (Mcal/kg)		
Gross energy	4.51	4.52
Metabolizable energy	2.38	2.19

The total mixed ration was provided twice daily (0800 and 1600 h), and water was offered *ad libitum*. The experiment lasted 180 days.

### Dry matter intake, body weight change, and feeding efficiency

Dry matter intake (DMI) and nutrient intake were obtained by the difference between each component offered in the diet and the total contained in the leftovers and expressed as grams per day (g/day). Daily intake was adjusted to allow 10% leftovers.

The animals were weighed every 15 days before the first feed supply of the day, and the last weighing was performed before the beginning of the pre-slaughter fasting. The average daily gain (ADG) was calculated as the quotient of the difference between the final BW and the initial BW by the duration in days on the feedlot for each age evaluated. The feed efficiency (FE) was obtained by the relationship between the ADG and DMI variables.

### Digestibility trial and collection of urine

This study had three collection periods of feeds, leftovers, feces, and urine. All collections were conducted during the last 5 days preceding each slaughter (A1, A2, and A3 days of age). Feed and leftovers were collected daily during the entire collection period before feeding the animals.

Feces were collected from the animals’ rectal ampulla at specific times (0800 h on the first day, 1200 h on the second day, and 1600 h on the third day), and a composite fecal sample from each animal was obtained at the end of each collection period from quantities proportional to those collected on each day and stored at − 20 °C for posterior analyses (Costa et al. 2013). To estimate fecal excretion, indigestible NDF (iNDF) was used as an internal indicator. The iNDF content in feeds, leftovers, and feces was obtained after 288 h of incubation of samples in the rumen of an adult cow fed a diet based on Tifton 85 grass hay and concentrate. Incubation was carried out using nylon bags with a porosity of 50 µm and a proportion of 15 mg of sample per cm^2^ of bag area (Detmann et al. 2023). After incubation, the bags were washed, subjected to NDF analysis (Van Soest et al. 1991), dried, and weighed and the residue remaining considered as iNDF. Fecal DM excretion was calculated as the ratio between iNDF intake and iNDF concentration in feces.

Urine collection was performed by spot sampling at 1200 h on the first day of fecal collection, 4 h post-feeding. Colostomy bags fixed to the vulva with wax were used as urine collectors by spontaneous urination. After urination, the bags were removed, and urine was filtered through gauze. Aliquots of 5 mL of urine from each animal were diluted with 45 mL of a solution of 0.018 N sulfuric acid (H_2_SO_4_) and stored at − 20 °C (Pereira et al. 2018) for analysis.

### Slaughtering procedures and sampling of body components

Before each slaughter (baseline group; A1, A2, and A2 ages), fasting body weight (FBW) was obtained after 18 h without feed and water (Pereira et al. 2018). Slaughter was carried out by stunning with a captive bolt pistol, followed by sectioning of the jugular vein and carotid artery, skinning, and evisceration. The blood was weighed and sampled. The hide was weighed, cut into strips, sampled, and frozen. The components of the gastrointestinal tract (GIT), bladder, and gallbladder were weighed full, washed, and after drained and weighed again. The individual components head, hooves, hide, heart, lung + trachea/tongue + esophagus combined, liver, pancreas, spleen, kidneys, mesenteric fat, omental fat, heart fat, perirenal fat, rumen reticulum, omasum, abomasum, small intestine, large intestine, bladder, reproductive tract, mammary gland, and the carcass were weighed separately. The carcasses were refrigerated at 4 °C for 24 h and then divided into right and left half carcasses.

Subsequently, the hide samples, right half carcasses, and all other non-carcass components (blood, internal organs, visceral fat, head, and hooves) from each animal were frozen separately at − 20 °C, cut with a band saw, and ground in an industrial cutter. After grinding and homogenization, samples were taken from the combined mass of the non-carcass components, the right half carcasses, and the hides, which were frozen at − 20 °C for subsequent chemical analyses.

### Laboratory analyses

Feeds, leftovers, and feces were pre-dried at 55 °C for 72 h in a forced-ventilation oven and ground in a knife mill with a 1-mm sieve (Wiley Mill, Thomas, A. H., Philadelphia, PA, USA). The samples were analyzed for the concentration of DM (AOAC 1990-method 967.03); mineral matter (Association of Official Analytical Chemists - AOAC 1990; method 942.05), crude protein (CP; AOAC 1990; method 981.10), and ether extract (AOAC 1990; method 920.39). The NDF content was determined according to Van Soest et al. (1991) with thermostable alpha-amylase, without sodium sulfite, and corrected for ash and protein (Licitra et al. 1996) and non-fibrous carbohydrates were calculated by Detmann et al. (2023).

The concentration of creatinine in spot urine was quantified by the alkaline picrate method (Henry et al. 1974) using a commercial kit (Labtest, Lagoa Santa, MG, Brazil) and a spectrophotometer. From the daily excretion and concentration of creatinine (mg/L) in the spot sample, the urinary volume was estimated (Santos et al. 2024).

The gross energy (GE; Mcal/kg) of feeds, leftovers, feces, and urine was measured in an adiabatic calorimetric bomb (Model IKA C200, Cincinnati, OH, USA). From the pre-dried and ground samples of feeds, leftovers, and feces, an aliquot of approximately 500 mg was taken, which was pressed, weighed, and subjected to combustion. For urine samples, pre-drying was initially carried out in a glass beaker at 55 °C in a forced ventilation oven until constant weight. Then, the pre-drying residue was placed in polyethylene capsules, weighed, and subjected to combustion (urine + capsule). The GE of urine was calculated by discounting the GE of the capsule, which was subjected to combustion alone.

Samples of body components (hide, right half carcasses, non-carcass components) were pre-dried at 55 °C in an oven with forced air circulation until constant weight. They were then defatted in ether for 12 h (Pereira et al. 2017) in a Soxhlet apparatus (AOAC 1990; method 920.39). The fat-free samples were ground in a ball mill and then analyzed for the concentration of DM (AOAC 1990; method 967.03) and CP (AOAC 1990; method 981.10).

### Calculations and adjusted equations

The EBW was calculated according to the equation:

$${\rm{EBW = FBW - }}\left( {{\rm{GIT \,content + Bladder\, content + Gallbladder \,content}}} \right)$$

where EBW is empty body weight (kg), FBW is fasting body weight (kg), and GIT corresponds to the gastrointestinal tract.

The metabolizable energy intake (MEI) was calculated according to the following equations:

$${\rm{GEI = }}\left( {{\rm{G}}{{\rm{E}}_{{\rm{roughage}}}}{\rm{ + G}}{{\rm{E}}_{{\rm{concentrate}}}}} \right){\rm{ - G}}{{\rm{E}}_{{\rm{leftovers}}}}$$

$${\rm{MEI = GEI - }}\left( {{\rm{G}}{{\rm{E}}_{{\rm{feces}}}}{\rm{ + G}}{{\rm{E}}_{{\rm{urine}}}}{\rm{ + G}}{{\rm{E}}_{{\rm{gases}}}}} \right)$$

where GEI is the gross energy intake (Mcal/day), DEI is the digestible energy intake (Mcal/day), and MEI corresponds to the metabolizable energy intake (Mcal/day). GE of gases was calculated using the equation proposed by Blaxter and Clapperton (1965), as follows:

$${\rm{G}}{{\rm{E}}_{{\rm{gases}}}}{\rm{ = GEI \times }}\left( {{\rm{4}}{\rm{.28 + 0}}{\rm{.059 \times DGE}}} \right)$$

where GE_gases_ is the gross energy of the gases (Mcal/day), GEI is the gross energy intake (Mcal/day), and DGE corresponds to the digestibility of the gross energy (%).

To estimate the relationship between BW and EBW, and between EBWG and ADG, linear regressions were adjusted as follows:

$${\rm{EBW = \beta 0 + \beta 1 \times BW}}$$

$${\rm{EBWG = \beta 0 + \beta 1 \times ADG}}$$

Where EBW is the empty body weight (kg), BW is the body weight (kg), EBWG is the empty body weight gain (kg/day), ADG corresponds to the average daily gain (kg/day), β_0_ and β_1_ are regression parameters.

To estimate the initial EBW of females submitted to the experimental test, information from the baseline group was used, considering that these animals were representative of all females at the beginning of the experiment. Thus, regression equations for FBW against BW and EBW against FBW were adjusted based on baseline female information. Likewise, the initial body energy of the experimental females was predicted by regression equations of the body energy content in relation to the EBW of the baseline females.

The body energy content was obtained from the body contents of protein and fat and their respective caloric equivalents, according to ARC (1980):

$${\rm{BE = }}\left( {{\rm{5}}{\rm{.64 \times BPC}}} \right){\rm{ + }}\left( {{\rm{9}}{\rm{.39 \times BFC}}} \right)$$

where BE is the body energy (Mcal), BPC is the body protein content (kg), and BFC is the body fat content (kg). The daily retained energy (RE; Mcal/day) of each animal was obtained as final RE minus initial RE divided by experimental days. The heat production (HP; Mcal/day) was obtained as follows: it was obtained from the differences between metabolizable energy intake (MEI) and RE.

$${\rm{HP = MEI - RE}}$$

where HP is the heat production (Mcal/day), MEI is the metabolizable energy intake (Mcal/day), and RE is the retained energy (Mcal/day).

To estimate the net energy requirements for maintenance (NE_m_), a non-linear exponential model (Ferrell and Jenkins 1998) was used to describe the relationship between HP (Mcal/kg^0.75^ EBW/day) and MEI (Mcal/kg^0.75^ EBW/day) as follows:

$${\rm{HP = \beta 0 \times }}{{\rm{e}}^{{\rm{\beta 1 \times MEI}}}}$$

where HP is the heat production (Mcal/kg^0.75^ EBW/day), MEI is the metabolizable energy intake (Mcal/kg^0.75^ EBW/day), *e* corresponds to the Euler number, β_0_ represents the NE_m_ (Mcal/kg^0.75^ EBW/day), and β1 is the slope of the exponential ratio.

For estimating the metabolizable energy requirements for maintenance (ME_m_), an iterative calculation process was applied to the previously presented model to determine the point where the MEI and HP are equal (i.e., the point at which there is no energy retention in the body). Thus, the efficiency of metabolizable energy utilization for maintenance (*k*_*m*_) was obtained as follows:

$$km = {\rm{NEm/MEm}}$$

To predict the net energy requirements for gain (NE_g_), the model suggested by the NRC (1981) was used, which describes the relationship between RE and EBWG for a given EBW:

$${\rm{RE = \beta 0 \times EB}}{{\rm{W}}^{{\rm{0}}{\rm{.75}}}}{\rm{ \times EBW}}{{\rm{G}}^{{\rm{\beta 1}}}}$$

where RE is the retained energy (Mcal/day), EBW^0.75^ is metabolic empty body weight (kg), EBWG is empty body weight gain (kg/day), β_0_ and β_1_ are the regression parameters. In this model, RE represents NE_g_.

To estimate the metabolizable energy requirements for gain (ME_g_), the efficiency of metabolizable energy utilization for gain (*k*_*g*_) was estimated from the linear regression of RE as a function of the MEI for gain (MEI_g_), according to Galvani et al. (2014):

$${\rm{RE = \beta 0 + \beta 1 \times ME}}{{\rm{I}}_{\rm{g}}}$$

where RE is the retained energy (Mcal/kg^0.75^ EBW/day), MEI_g_ is the metabolizable energy intake for gain (Mcal/kg^0.75^ EBW/day), β_0_ and β_1_ are regression parameters. MEI_g_ was calculated for each animal by the difference between MEI and ME_m_. Thus, ME_g_ was calculated as follows:

$${\rm{M}}{{\rm{E}}_{\rm{g}}}{\rm{ = N}}{{\rm{E}}_{\rm{g}}}{\rm{/}}{k_g}$$

where ME_g_ is the metabolizable energy requirements for gain (Mcal/kg^0.75^ EBW/day), NE_g_ is the net energy requirements for gain (Mcal/kg^0.75^ EBW/day), and *k*_*g*_ is the efficiency of metabolizable energy utilization for gain.

The partial efficiencies of metabolizable energy utilization for fat (*k*_*fat*_) and protein (*k*_*prot*_) synthesis were estimated using a multiple regression between MEI and RE as fat and protein (Rattray et al. 1974):

$${\rm{MEI = \beta 0 + \beta 1 \times R}}{{\rm{E}}_{{\rm{fat}}}}{\rm{ + \beta 2 \times R}}{{\rm{E}}_{{\rm{prot}}}}$$

where MEI is the metabolizable energy intake (Mcal/kg^0.75^ EBW/day), RE_fat_ is the retained energy as fat (Mcal/kg^0.75^ EBW/day), RE_prot_ is the retained energy as protein (Mcal/kg^0.75^ EBW/day), β_0_ represents ME_m_, and the coefficients β_1_ and β_2_ correspond to the amounts of ME necessary to deposit 1 Mcal of ME as fat and protein, respectively. The partial efficiencies *k*_*fat*_ and *k*_*prot*_ were calculated as the inverse of the coefficients β_1_ and β_2_, respectively.

Dietary requirements were calculated from the sum of energy requirements for weight maintenance and weight gain. The DE and total digestible nutrients (TDN) requirements were calculated considering that ME corresponds to 85% of DE (Brito Neto et al. 2023) and 1 kg of TDN contains 4.409 Mcal of DE.

### Statistical analysis

The assumptions of normality and homogeneity of variance were verified using the Shapiro-Wilk and Levene tests, respectively. Subsequently, all analyses were performed using SAS Software (SAS 9.4, SAS Institute Inc., Cary, NC, USA). The variables studied related to intake, digestibility, performance, body composition, and energy flow were analyzed using the GLM procedure in SAS software version 9.2 (SAS Inst. Inc., Cary, NC), assuming a completely randomized design in a factorial arrangement, according to the following mathematical model: Y_ijk_ = μ + A_i_ + C_j_ + A_i_ × C_j_ + ε_ijk_, where: Y_ijk_ is the dependent or response variable measured in animal or experimental unit “k,” at age “i,” with concentrate level “j”; μ is the population mean or global constant; A_i_ is the effect of age “i”; C_j_ is the effect of concentrate level “j”; A_i_ × C_j_ is the interaction between the effects of age “i” and concentrate level “j”; and ε_ijk_ represents the unobserved random error. The Tukey test was used for comparisons, considering *p* < 0.05 to determine statistical significance between the treatment means. Animals fed at maintenance levels were included only in the mean comparisons related to energy flow, since these animals do not show productive responses comparable to those of animals fed *ad libitum*. For estimation of energy requirements, linear models were fitted using the PROC MIXED procedure, and the nonlinear models were fitted with PROC NLMIXED. The Ordinary Least Squares method was employed for linear models, whereas nonlinear models were adjusted using the Gauss-Newton algorithm. In all models, the effect of age was examined on both parameters, β_0_ and β_1_. Statistical significance was determined at *p* < 0.05.

## Results

## Intake and digestibility

Increasing age resulted in higher intakes (*p* ≤ 0.001) of DM, CP, organic matter, and NFC in females in age A3 compared to other age groups, while NDFap intake remained relatively constant across ages (Table 3). Females fed a CL1 exhibited higher (*p* < 0.001) intake of NFC, whereas those fed a CL2 showed greater (*p* < 0.001) NDFap intake. No effect of age was observed on the apparent digestibility of the evaluated components (*p* ≥ 0.078). On the other hand, the digestibility coefficients of DM, OM, and NFC were higher (*p* ≤ 0.001) in females fed the CL1, while CP digestibility was greater (*p* = 0.010) in those fed the CL2 MEI was influenced by age (*p* = 0.003), being higher in females in age A3 compared to the other age groups.

**Table 3:** Intake and apparent digestibility of dry matter and nutrients in Santa Ines females fed different concentrate levels at increasing ages

Item	A1	A2	A3		CL1	CL2	SEM	^**a**^*p*-value		
								A	CL	A × CL
Intake (g/day)										
DM	803.81^b^	739.77^b^	912.23ª		807.84	829.37	27.44	0.001	0.496	0.484
CP	116.07^b^	104.64^b^	131.37ª		116.55	118.17	3.87	0.001	0.714	0.267
OM	749.58^b^	688.62^b^	850.02ª		762.19	763.29	25.45	0.001	0.970	0.453
EE	24.16^b^	21.93^b^	27.67ª		26.81^a^	22.36^b^	0.813	< 0.001	< 0.001	0.103
NDF_ap_	374.89^ab^	358.05^b^	428.26ª		324.08^b^	450.05^a^	14.56	0.008	< 0.001	0.815
NFC	234.46^b^	204.01^b^	262.72^a^		294.75^a^	172.71^b^	7.96	< 0.001	< 0.001	0.066
MEI (Mcal/day)	1.80^b^	1.71^b^	2.09^a^		1.91	1.82	0.068	0.003	0.261	0.780
Digestibility (g/kg DM)										
DM	619.62	650.85	636.21		665.84^a^	605.29^b^	12.26	0.206	0.001	0.403
CP	631.94	661.27	655.81		622.80^b^	676.55^a^	16.26	0.404	0.010	0.163
OM	635.82	666.00	653.01		683.86^a^	619.35^b^	12.18	0.222	< 0.001	0.500
NDF_ap_	542.09	594.83	565.80		563.48	571.67	15.88	0.078	0.654	0.585
NFC	787.40	792.56	789.02		849.58^a^	729.75^b^	10.06	0.928	< 0.001	0.595

A1 = 200 days of age; A2 = 250 days of age; A3 = (330 days of age); CL1 = 50% concentrate  < i icoretag=“italic” isexistfmt=“true” aid=“54s730i16yav28x” ia_version=“0” > ad libitum; CL12 = 25% concentrate  < i icoretag=“italic” isexistfmt=“true” aid=“yv7i162sa0x5438” ia_version=“0” > ad libitum; CL2R = 25% concentrate with restricted intake at 1.9% of body weight; DM = dry matter; CP = crude protein; OM = organic matter; EE = ether extract; NDF < sub icoretag=“sub” isexistfmt=“true” aid=“1405x7a8s6yv23i” ia_version=“0” > ap  = neutral detergent fiber corrected for ash and protein; NFC = non-fibre carbohydrates; MEI = metabolizable energy intake; SEM = standard error of the mean.  < sup icoretag=“sup” isexistfmt=“true” aid=“041y6837isaxv52” ia_version=“0” > a Means followed by a different letter in the row are statistically different according to the Tukey’s test at 5% probability

## Energy flow

**Fig. 2:**
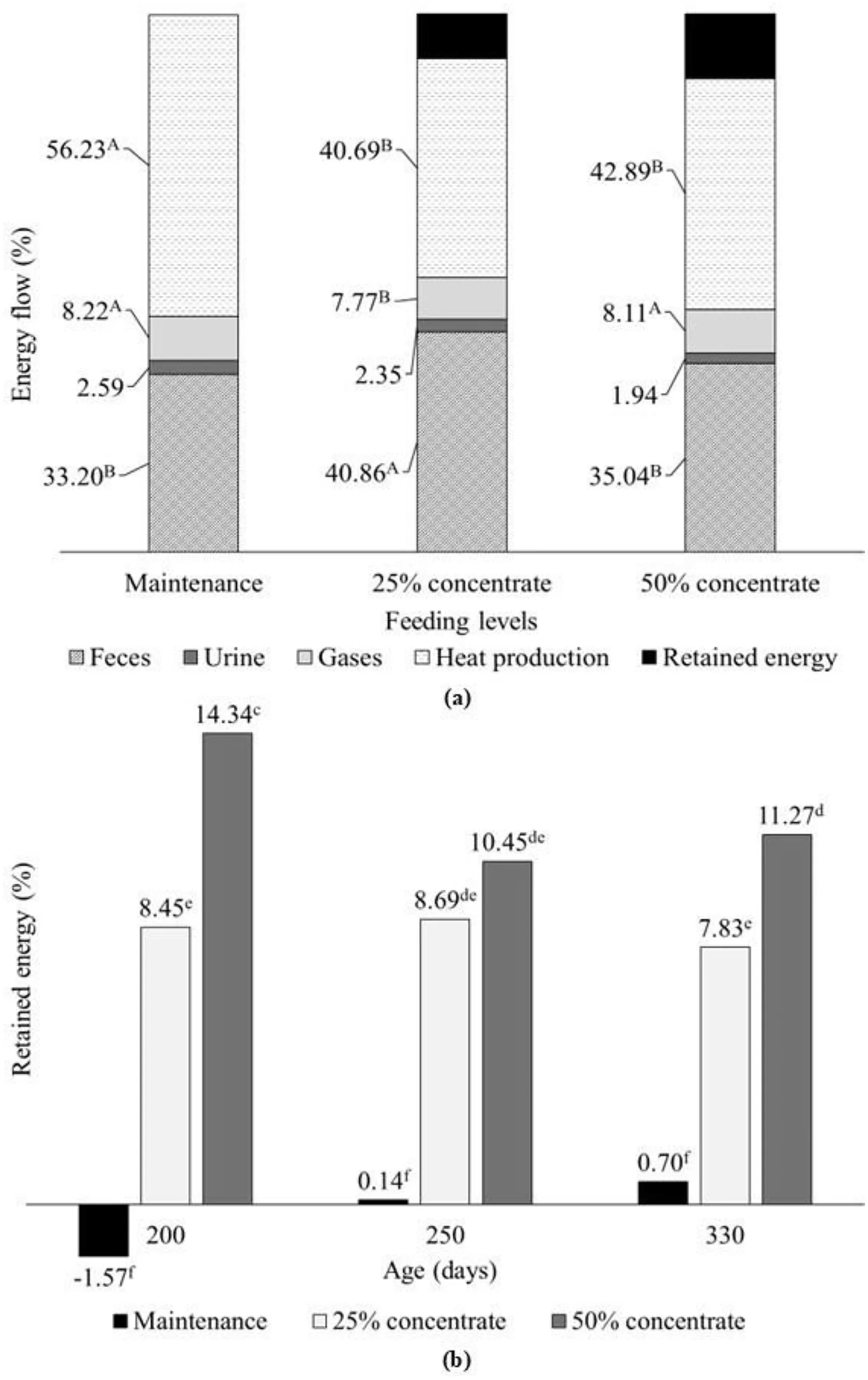
Energy flow (%) of hair sheep females at different feeding levels (**a**) and effect of the interaction between ages and feeding levels on retained energy (**b**). Uppercase letters A, B indicate differences between feeding levels (*p* < 0.05). Lowercase letters c-f indicate differences between all treatments (*p* < 0.05)

Considering the energy flow as a percentage of the GEI, no effect (*p* > 0.164) of age was verified (Fig. 2). GE_feces_, GE_gases_ and HP were affected (*p* < 0.001) by concentrate levels, with GE_feces_ being higher for females receiving CL2, GE_gases_ being similar and higher for the CL1 and maintenance feeding levels, and HP being higher for the maintenance level. GE_urine_ was similar between feeding levels (*p* = 0.111). The percentage of RE, in turn, was affected (*p* = 0.035) by the interaction between slaughter age and concentrate levels.

## Performance and body composition

Final BW, FBW, and EBW varied (*p* < 0.001) among the evaluated ages (Table 4). However, EBW was also influenced by concentrate levels, being higher (*p* = 0.015) in females fed a 50% concentrate diet. ADG was greater (*p* = 0.003) in younger females (A1) compared to older ones. The EBWG was influenced by the interaction (*p* = 0.021) between age and concentrate levels. Both age (*p* = 0.001) and concentrate levels (*p* = 0.001) affected the carcass yield (CY), which was higher in females in age A3 and in those fed a CL1. Age significantly influenced (*p* < 0.001) visceral organ mass (VOM), which was greater in females in age A3. Visceral fat (VF) varied according to the interaction (*p* = 0.006) between age and concentrate levels. FE decreased (*p* < 0.001) with increasing age, being higher in females in age A1. Energy concentration of gain (ECg) increased with age (*p* = 0.053), and was higher (*p* = 0.021) in females fed a CL1. Regarding body composition, both age and concentrate levels affected the percentage of water (*p* ≤ 0.009) and EE (*p* ≤ 0.001), as well as energy (*p* ≤ 0.001; Mcal/kg) in EBW. Water content decreased at A3 age and was higher in females fed CL2. On the other hand, EE and energy content were higher in older females and in those fed CL1. There was no effect (*p* ≥ 0.116) of age and concentrate levels on CP content. MM content was affected only by concentrate levels (*p* < 0.001), being higher in animals that ingested CL2.

**Table 4:** Performance and body composition of Santa Ines females fed different concentrate levels at increasing ages

Item	A1	A2	A3	CL1	CL2	SEM	^a^*p*-value		
							A	CL	A × CL
Initial BW (kg)	16.13	15.93	16.61	16.21	16.23				
Final BW (kg)	23.49^b^	26.86^b^	36.18^a^	29.04	28.64	1.04	<0.001	0.747	0.501
FBW (kg)	21.46^b^	24.93^b^	34.30^a^	27.38	26.41	1.01	<0.001	0.415	0.483
EBW (kg)	17.25^c^	20.06^b^	28.96^a^	23.25^a^	20.93^b^	0.753	<0.001	0.015	0.251
ADG (kg/day)	0.147^a^	0.109^b^	0.109^b^	0.124	0.119	0.008	0.003	0.559	0.059
EBWG (kg/day)	0.121	0.090	0.097	0.114	0.090	0.005	0.001	0.001	0.021
CY (%)	43.21^b^	45.55^b^	48.52^a^	47.74^a^	43.77^b^	0.822	0.001	0.001	0.942
VOM (kg)	2.72^b^	2.91^b^	3.49^a^	3.07	3.01	0.106	<0.001	0.676	0.437
VF (kg)	0.646	0.949	2.20	1.53	1.00	0.126	<0.001	0.002	0.006
FE	0.180^a^	0.146^b^	0.122^b^	0.152	0.147	0.008	<0.001	0.617	0.168
EC_g_ (Mcal/kg EBWG)	3.34^b^	3.50^ab^	3.85^a^	3.77^a^	3.36^b^	0.140	0.053	0.021	0.528
Body composition									
Water (% EBW)	64.41^a^	62.44^a^	56.83^b^	60.11^b^	62.33^a^	0.662	<0.001	0.009	0.784
CP (% EBW)	16.56	16.54	16.43	16.33	16.70	0.197	0.876	0.116	0.543
EE (% EBW)	14.69^b^	16.72^b^	22.56^a^	19.56^a^	16.41^b^	0.701	<0.001	0.001	0.491
MM (% EBW)	3.42	3.29	3.32	3.07^b^	3.61^a^	0.104	0.635	<0.001	0.252
Energy (Mcal/kg EBW)	2.31^b^	2.50^b^	3.05^a^	2.76^a^	2.48^b^	0.063	<0.001	0.001	0.576

A1 = 200 days of age; A2 = 250 days of age; A3 = (330 days of age); CL1 = 50% concentrate  < i icoretag=“italic” isexistfmt=“true” aid=“5x3ay0s728iv416” ia_version=“0” > ad libitum; CL12 = 25% concentrate  < i icoretag=“italic” isexistfmt=“true” aid=“26asx3y17i8450v” ia_version=“0” > ad libitum; CL2R = 25% concentrate with restricted intake at 1.9% of body weight; BW = body weight; FBW = fasting body weight; EBW = empty body weight; ADG = average daily gain; EBWG = empty body weight gain; CY = carcass yield; VOM = visceral organ mass; VF = visceral fat; FE = feed efficiency; EC < sub icoretag=“sub” isexistfmt=“true” aid=“x5v1y6478s320ia” ia_version=“0” > g  = energy concentration of gain; CP = crude protein; EE = ether extract; MM = mineral matter; SEM = standard error of the mean;  < sup icoretag=“sup” isexistfmt=“true” aid=“34x62vi158yas70” ia_version=“0” > a Means followed by a different letter in the row are statistically different according to Tukey’s test at 5% probability

## Energy requirements

The descriptive statistics of the Santa Ines female database generated in this study and used to adjust the equations and estimate energy requirements are shown in Table 5. Age did not influence the intercept (*p* = 0.317) and slope (*p* = 0.128) of the linear regression between EBW and BW, and a single equation was adjusted to describe the relationship. Likewise, there was no significant effect of age on the intercept (*p* = 0.697) and slope (*p* = 0.248) of the linear regression between EBWG and ADG, with a single equation being adjusted, as follows:

$${\rm{EBW}}\left( {{\rm{kg}}} \right){\rm{ = - 2}}{\rm{.393}}\left( {{\rm{ \pm 0}}{\rm{.646}}} \right){\rm{ + 0}}{\rm{.853}}\left( {{\rm{ \pm 0}}{\rm{.025}}} \right){\rm{ \times BW}}$$

$${{\rm{R}}^{\rm{2}}}{\rm{ = 0}}{\rm{.97;RMSE = 1}}{\rm{.203}}$$

$${\rm{EBWG}}\left( {{\rm{kg}}} \right){\rm{ = 0}}{\rm{.006}}\left( {{\rm{ \pm 0}}{\rm{.003}}} \right){\rm{ + 0}}{\rm{.782}}\left( {{\rm{ \pm 0}}{\rm{.032}}} \right){\rm{ \times ADG}}$$

$${{\rm{R}}^{\rm{2}}}{\rm{ = 0}}{\rm{.95;RMSE = 0}}{\rm{.013}}$$

**Table 5:** Description of the dataset of Santa Ines females at different ages used to estimate energy requirements

Item	A1 n = 12	A2 n = 12	A3 n = 12
Initial BW (kg)			
Minimum	14.16	13.56	14.26
Maximum	19.32	18.88	18.94
Final BW (kg)			
Minimum	14.68	14.42	14.86
Maximum	24.80	32.28	42.06
Initial FBW (kg)			
Minimum	13.11	12.53	13.20
Maximum	18.10	17.67	17.73
Final FBW (kg)			
Minimum	12.90	13.30	13.66
Maximum	22.82	29.54	40.00
Initial EBW (kg)			
Minimum	9.81	9.38	9.88
Maximum	13.48	13.17	13.21
Final EBW (kg)			
Minimum	10.19	9.64	9.83
Maximum	18.70	24.08	35.27
ADG (kg)			
Minimum	-0.038	-0.021	-0.007
Maximum	0.180	0.168	0.140
EBWG (kg)			
Minimum	-0.014	-0.011	-0.005
Maximum	0.147	0.114	0.131
MEI (Mcal/kg^0.75^EBW/day)			
Minimum	0.110	0.106	0.112
Maximum	0.306	0.252	0.248
HP (Mcal/kg^0.75^EBW/day)			
Minimum	0.116	0.111	0.109
Maximum	0.244	0.207	0.209
RE (Mcal/kg^0.75^EBW/day)			
Minimum	-0.009	-0.005	-0.001
Maximum	0.075	0.052	0.053
RE (Mcal/day)			
Minimum	-0.051	-0.035	-0.003
Maximum	0.565	0.387	0.565
RE_fat_(Mcal/kg^0.75^EBW/day)			
Minimum	-0.010	-0.005	-0.0001
Maximum	0.061	0.041	0.043
RE_protein_ (Mcal/kg^0.75^EBW/day)			
Minimum	-0.0002	-0.002	-0.001
Maximum	0.018	0.012	0.011

A1 = 200 days of age; A2 = 250 days of age; A3 = (330 days of age); BW = body weight; FBW = fasting body weight; EBW = empty body weight; ADG = average daily gain; EBWG = empty body weight gain; MEI = metabolizable energy intake; HP = heat production; RE = retained energy

**Fig. 3:**
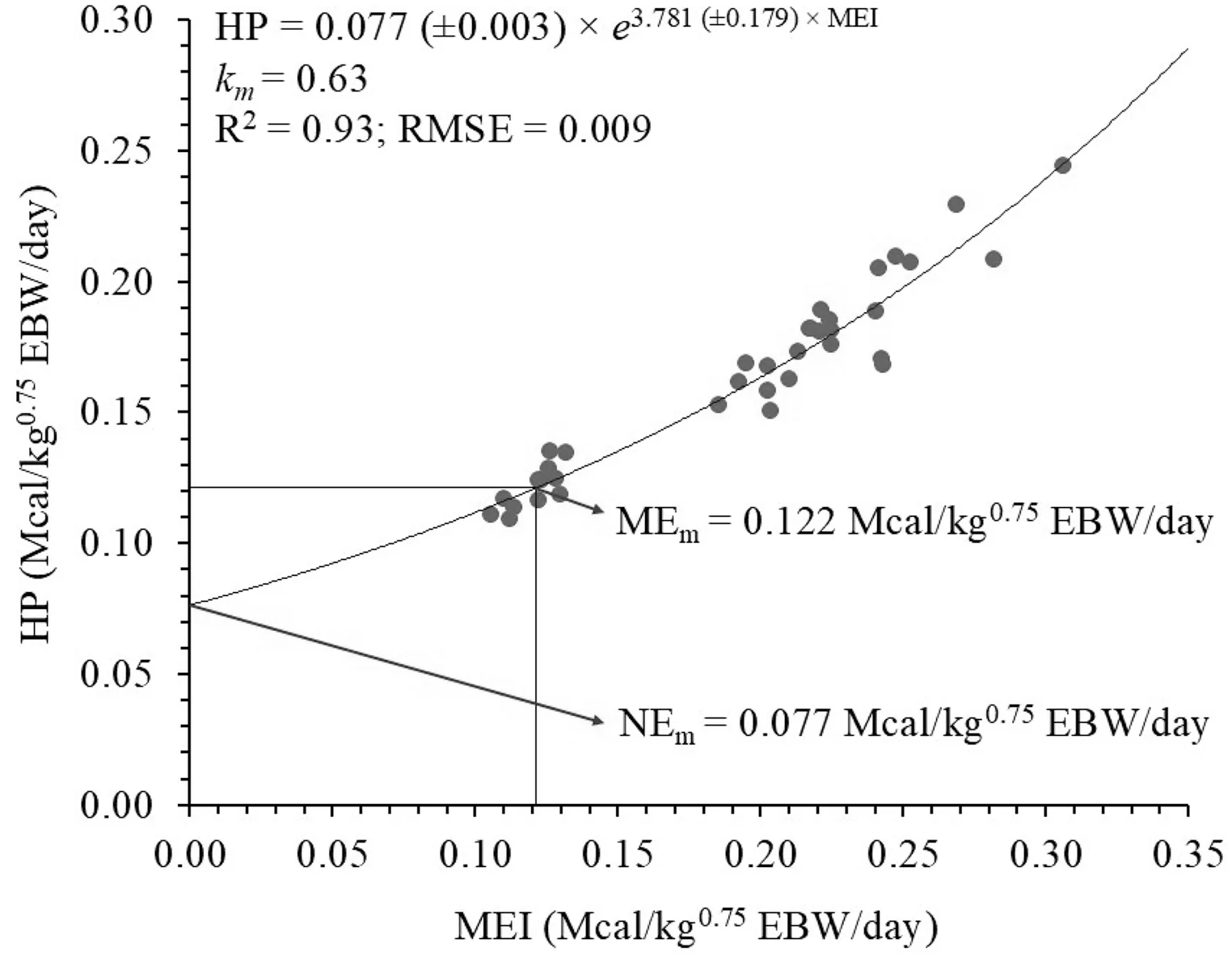
Relationship between heat production (HP) and metabolizable energy intake (MEI) of Santa Ines females used to estimate net (NE_m_) and metabolizable (ME_m_) energy requirements for maintenance

The intercept of the exponential relationship between HP and MEI corresponds to NE_m_ (Mcal/kg^0.75^ EBW/day), that is, the HP mathematically extrapolated to MEI equal to zero. The intercept (*p* = 0.190) and slope (*p* = 0.134) of this relationship were not significantly influenced by the ages studied, and a single equation was adjusted (Fig. 3):

$$\eqalign{{\rm{HP}}\left( {{\rm{Mcal/k}}{{\rm{g}}^{{\rm{0}}{\rm{.75}}}}{\rm{EBW/day}}} \right){\rm{ = }} & {\rm{ 0}}{\rm{.077}}\left( {{\rm{ \pm 0}}{\rm{.003}}} \right) \cr & {\rm{ \times }}{{\rm{e}}^{{\rm{3}}{\rm{.781}}\left( {{\rm{ \pm 0}}{\rm{.179}}} \right){\rm{ \times MEI}}}} \cr}$$

$${{\rm{R}}^{\rm{2}}}{\rm{ = 0}}{\rm{.93;RMSE = 0}}{\rm{.009}}$$

NE_m_ was estimated at 0.077 Mcal/kg^0.75^ EBW/day. Considering the point at which HP equaled MEI, ME_m_ was estimated in the present study at 0.122 Mcal/kg^0.75^ EBW/day. The relationship between NE_m_ and ME_m_, *k*_*m*_ was obtained as 0.63.

**Fig. 4:**
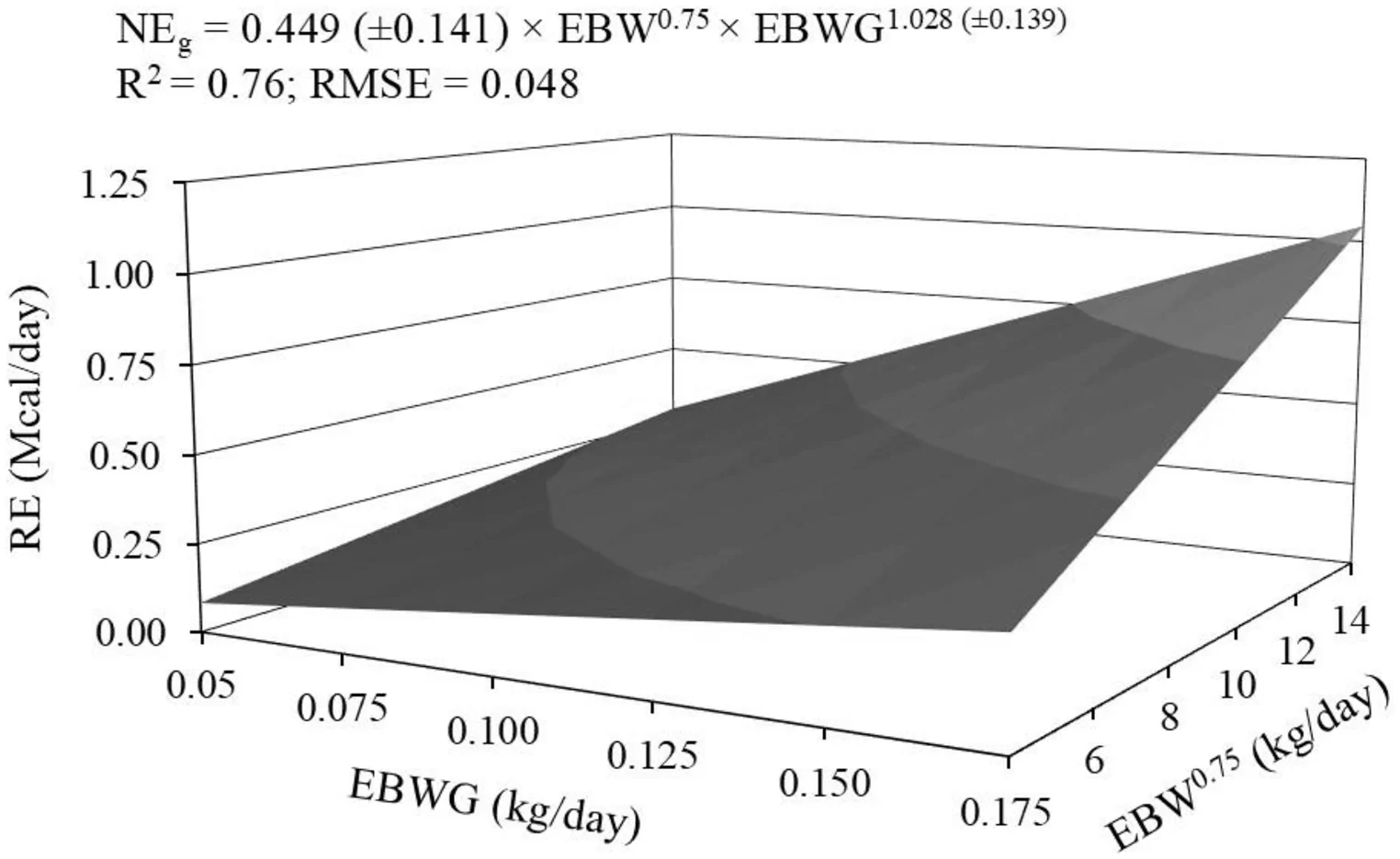
Relationship between retained energy (RE) and empty body weight gain (EBWG) for a given empty body weight (EBW) of hair sheep females used to estimate energy requirements for gain (NE_g_)

The relationship between RE as a function of EBW^0.75^ and EBWG, where RE corresponds to NE_g_, was not significantly influenced by age (*p* = 0.521 for intercept; *p* = 0.520 for slope), resulting in a general equation (Fig. 4), as follows:

$$\eqalign{{\rm{N}}{{\rm{E}}_{\rm{g}}}\left( {{\rm{Mcal/day}}} \right){\rm{ = }} & {\rm{0}}{\rm{.449}}\left( {{\rm{ \pm 0}}{\rm{.141}}} \right){\rm{ \times EB}}{{\rm{W}}^{{\rm{0}}{\rm{.75}}}}{\rm{ }} \cr & {\rm{ \times EBW}}{{\rm{G}}^{{\rm{1}}{\rm{.028}}\left( {{\rm{ \pm 0}}{\rm{.139}}} \right)}} \cr}$$

$${{\rm{R}}^{\rm{2}}}{\rm{ = 0}}{\rm{.76;RMSE = 0}}{\rm{.048}}$$

**Fig. 5:**
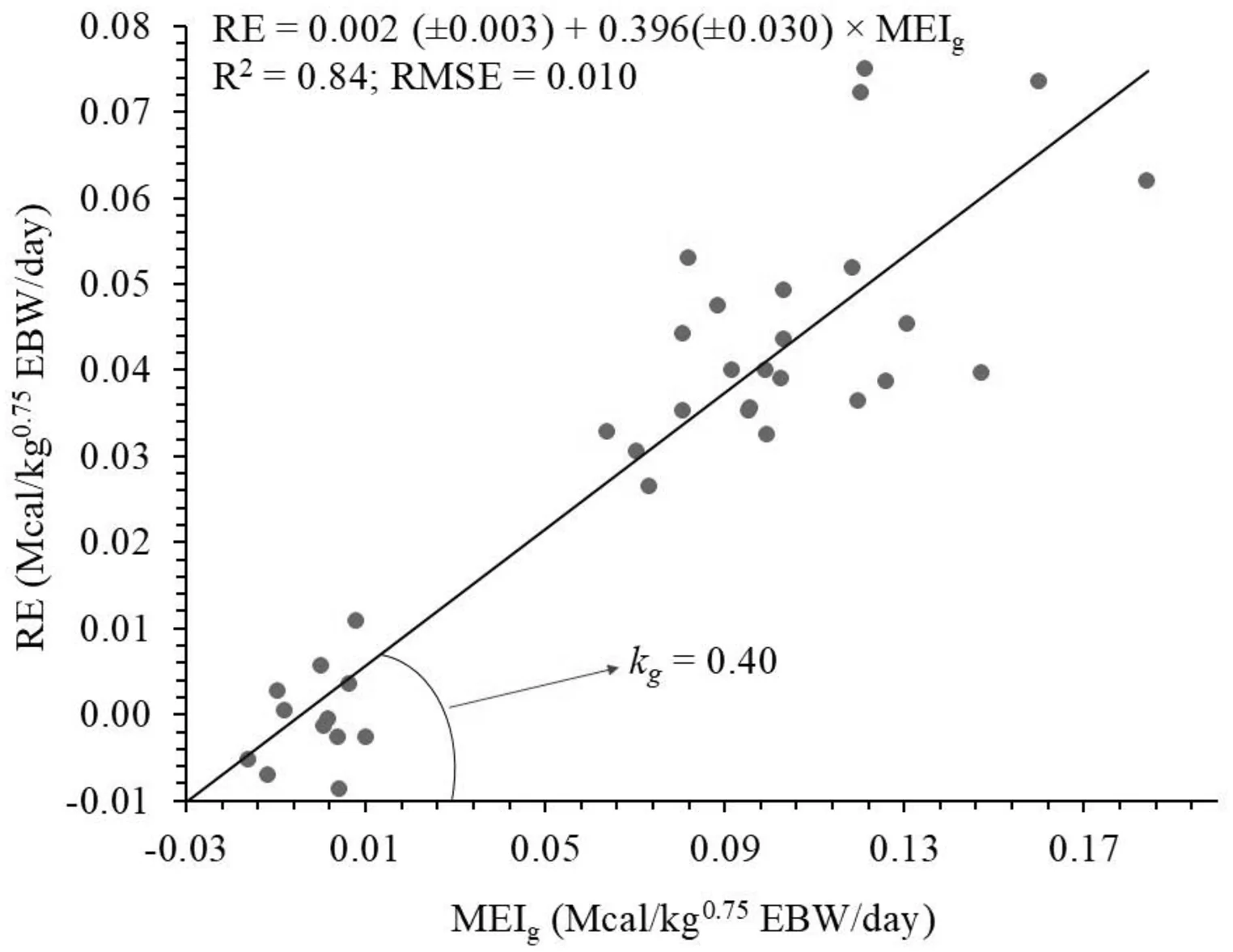
Relationship between retained energy (RE) and metabolizable energy intake for gain (MEI_g_) of Santa Ines females used to estimate the efficiency of utilization metabolizable energy for gain (*k*_*g*_)

The intercept (*p* = 0.804) and slope (*p* = 0.842) of the linear regression of RE as a function of MEIg were not significantly influenced by age, and a single equation was adjusted, where the slope corresponds to *k*_*g*_ (Fig. 5). The *k*_*g*_ in this study was estimated at 0.40, and the ME_g_ (Mcal/day) can be calculated by dividing the NE_g_ by 0.40. Regarding the partial efficiencies of using ME, age did not significantly influence the intercept (*p* = 0.610) and the slopes corresponding to RE_fat_ (*p* = 0.912) and RE_prot_ (*p* = 0.869) from the multiple regression of MEI as a function of RE as fat and protein. From this multiple regression, ME_m_ was estimated at 0.121 Mcal/kg^0.75^ EBW/day, and k_prot_ and k_gord_ corresponded to 0.16 and 0.78, respectively.

$$\eqalign{{\rm{RE}}\left( {{\rm{Mcal/k}}{{\rm{g}}^{{\rm{0}}{\rm{.75}}}}{\rm{EBW/day}}} \right){\rm{ = }} & {\rm{0}}{\rm{.002}}\left( {{\rm{ \pm 0}}{\rm{.003}}} \right) \cr & {\rm{ + 0}}{\rm{.396}}\left( {{\rm{ \pm 0}}{\rm{.030}}} \right){\rm{ \times ME}}{{\rm{I}}_{\rm{g}}} \cr}$$

$${{\rm{R}}^{\rm{2}}}{\rm{ = 0}}{\rm{.84;RMSE = 0}}{\rm{.010}}$$

$$\eqalign{{\rm{MEI}}\left( {{\rm{Mcal/k}}{{\rm{g}}^{{\rm{0}}{\rm{.75}}}}{\rm{EBW/day}}} \right){\rm{ = }} & {\rm{0}}{\rm{.121}}\left( {{\rm{ \pm 0}}{\rm{.005}}} \right) \cr & {\rm{ + 1}}{\rm{.284}}\left( {{\rm{ \pm 0}}{\rm{.536}}} \right){\rm{ \times R}}{{\rm{E}}_{{\rm{fat}}}} \cr & {\rm{ + 6}}{\rm{.093}}\left( {{\rm{ \pm 1}}{\rm{.619}}} \right){\rm{ \times R}}{{\rm{E}}_{{\rm{prot}}}} \cr}$$

$${{\rm{R}}^{\rm{2}}}{\rm{ = 0}}{\rm{.91;RMSE = 0}}{\rm{.017}}$$

The estimated energy requirements for maintenance, weight gain, and dietary of growing Santa Ines females were shown in Table 6.

**Table 6:** Dietary energy requirements of growing Santa Ines females

BW (kg)	EBW (kg)	ADG (kg)	EBWG (kg)	**NE**_**m**_**(Mcal/day)**	**ME**_**m**_**(Mcal/day)**	**NE**_**g**_**(Mcal/day)**	**ME**_**g**_**(Mcal/day)**	TME (Mcal/day)	DE (Mcal/day)	TDN (kg/day)
10	6.13	0.100	0.084	0.300	0.475	0.137	0.344	0.819	0.964	0.219
10	6.13	0.150	0.123	0.300	0.475	0.203	0.509	0.984	1.158	0.263
10	6.13	0.200	0.163	0.300	0.475	0.270	0.675	1.151	1.354	0.307
15	10.40	0.100	0.084	0.445	0.706	0.204	0.511	1.217	1.432	0.325
15	10.40	0.150	0.123	0.445	0.706	0.302	0.756	1.462	1.720	0.390
15	10.40	0.200	0.163	0.445	0.706	0.401	1.003	1.709	2.011	0.456
20	14.66	0.100	0.084	0.576	0.914	0.264	0.661	1.575	1.853	0.420
20	14.66	0.150	0.123	0.576	0.914	0.391	0.978	1.892	2.226	0.505
20	14.66	0.200	0.163	0.576	0.914	0.519	1.298	2.212	2.602	0.590
25	18.92	0.100	0.084	0.697	1.107	0.320	0.800	1.907	2.244	0.509
25	18.92	0.150	0.123	0.697	1.107	0.474	1.184	2.291	2.695	0.611
25	18.92	0.200	0.163	0.697	1.107	0.629	1.571	2.678	3.151	0.715
30	23.19	0.100	0.084	0.812	1.289	0.373	0.932	2.221	2.613	0.593
30	23.19	0.150	0.123	0.812	1.289	0.552	1.379	2.668	3.139	0.712
30	23.19	0.200	0.163	0.812	1.289	0.732	1.830	3.119	3.670	0.832
35	27.45	0.100	0.084	0.922	1.463	0.423	1.058	2.521	2.965	0.673
35	27.45	0.150	0.123	0.922	1.463	0.626	1.565	3.028	3.562	0.808
35	27.45	0.200	0.163	0.922	1.463	0.831	2.077	3.540	4.165	0.945
40	31.71	0.100	0.084	1.027	1.630	0.471	1.179	2.809	3.305	0.750
40	31.71	0.150	0.123	1.027	1.630	0.698	1.744	3.374	3.970	0.900
40	31.71	0.200	0.163	1.027	1.630	0.926	2.315	3.945	4.641	1.053

BW = body weight; EBW = empty body weight; ADG = average daily gain; EBWG = empty body weight gain; NEm = net energy requirements for maintenance; MEm = metabolizable energy requirements for maintenance; NEg = net energy requirements for gain; MEg = metabolizable energy requirements for gain; TME = total metabolizable energy; DE = digestible energy; TDN = total digestible nutrients

## Discussion

Dry matter intake is an important variable because it defines the amount of nutrients ingested and, consequently, determines animal performance (Van Soest 1994). Dry matter intake is mainly regulated by physical factors related to the distensibility capacity of the animal’s gastrointestinal tract (GIT), as well as physiological factors regulated by the energy available in the diet (Mertens 1994). In this study, dry matter intake (DMI) and metabolizable energy intake (MEI) did not vary between ad libitum concentrate levels, indicating the possibility of intake regulation by metabolic demand in diets with higher concentrate levels and by physical demand in diets with higher roughage content.

Regarding the digestibility of nutrients, this is related to the feed itself, rather than varying according to the animal’s characteristics (Coelho da Silva and Leão 1979). This fact explains the higher digestibility coefficients in diets with higher levels of concentrate, as well as the fact that age did not influence the digestibility of diets.

Body composition varies mainly with sex, feed intake, and age (NRC 2007; CSIRO 2007). Thus, when the animal is young, the composition of weight gain has higher proportions of protein and water; however, as the animal grows and its weight approaches maturity weight, the concentration of fat in weight gain increases, and protein and minerals stabilize in the animal’s empty body (BR-Caprinos & Ovinos 2024). Furthermore, fat deposition can be modulated by the level of energy intake. In this study, at age A3, an increase in energy concentration of gain (Mcal/kg EBWG), CY (%), and VOM (kg) was observed, along with greater visceral fat accumulation, particularly with dietary level CL1. In contrast, with the increase in age, FE declines, as shown by lower ADG. The ADG (kg/day) is influenced by gain composition, with greater gains reflecting higher protein deposition (Owens et al. 1995), notably in females with A2 age fed the level of concentrate CL1.

Despite the variation in body composition, no age effect was observed in energy flow expressed as a percentage of GEI, which explains the absence of an age effect in NEm. Expressing energy flow in percentage allows understanding metabolic priorities under different conditions (Brito Neto et al. 2023). Differences were observed in energy flow (%) between feeding levels. This can be associated with the law of diminishing returns, in which small increases in the feeding plan above maintenance result in a compression of HP (%) and a greater energy supply for body retention (VandeHaar and St-Pierre 2006). These differences are important in estimating energy requirements in comparative slaughter studies (Lofgreen and Garrett 1968).

In a situation of ingestion equal to zero, there is no caloric increase, and the components of HP are the heat from voluntary activities and the heat from basal metabolism (NRC 1981), which correspond to NE_m_. In the present study, the NE_m_ was 0.077 Mcal/kg^0.75^ EBW/day (0.064 and 0.061 Mcal/kg^0.75^ FBW and BW/day, respectively). It has long been considered that fasting heat production per unit of metabolic size decreases as animals age (Blaxter 1962). However, we found that at 330 days, there is no variation in the NE_m_ of Santa Ines females.

Different responses to net energy requirements are due to the methodology used (comparative slaughter or calorimetry). Methodology plays a crucial role in maintenance values and the difference in values is linked to support metabolism (Williams and Jenkins 2003). Support metabolism increases energy demand when the animal transitions from a maintenance state to a growth or production state. When the breathing chamber is applied, generally observe a higher Nem. Therefore, support metabolism is accounted for in maintenance and observe greater efficiency in the use of metabolizable energy for gain (*k*_g_), consequently, lower ME_g_. However, when the comparative slaughter technique is used, generally observe a lower NEm, as well as a lower kg, and therefore, support metabolism is accounted for in the requirements for gain. Furthermore, differences in substrate cycles determine much of the variation in maintenance requirements. Protein recycling rates are higher in young animals and respond to low feeding levels, while triglyceride recycling increases in response to high feeding levels. Ion transport is greater in young animals compared to adults. Maintaining body temperature also contributes to energy expenditure for maintenance

The NEm value this study was 64 kcal/kg^0.75^ FBW, this average value was similar than the value reported by (Nie et al. 2015) (62 kcal/kg^0.75^ FBW) obtained from a comparative slaughter trial. The NE_m_ obtained in this study is close to the values recommended by the (NRC 2007) and (CSIRO 2007), of 0.062 and 0.066 Mcal/kg^0.75^ FBW/day, respectively, and lower than the (INRA 2018) recommendation of 0.074 Mcal/kg^0.75^ BW/day. When converting NE_m_ to ME_m_, a value of 0.122 Mcal/kg^0.75^ EBW/day (0.102 and 0.096 Mcal/kg^0.75^ FBW and BW/day, respectively) is obtained, which represents the energy required to maintain a stable physiological state, the exact balance between energy input and output, i.e. where there is no net change in the body’s energy content and energy intake is adequate to sustain zero energy balance (Baldwin 1995). A similar value (0.121 Mcal/kg^0.75^ EBW/day) was obtained from the multiple regression of MEI as a function of RE_fat_ and RE_prot_, which was also not affected by age. Close value was reported by Deng et al. (2014) for females ½ Dorper × ½ thin-tailed Han in growth (0.104 Mcal/kg^0.75^ FBW/day), lower by NRC (2007) (0.097 Mcal/kg^0.75^ FBW/day), and higher by Chay-Canul et al. (2011) for Pelibuey females (0.102 Mcal/kg^0.75^ BW/day). The variations observed in ME_m_ can be attributed to differences in *k*_*m*_, which can vary according to age, sex, genotype, body composition, and previous nutritional plan, as explained by Garrett (1980). In the present study, a *k*_*m*_ of 0.63 was obtained.

Typically, tropical genotypes ingest more fibrous feeds, which is associated with an increase in HP due to increased visceral energy expenditure, and this reduces *k*_*m*_ (Salah et al. 2014). However, the *k*_*m*_ obtained in this study is close to that suggested by the NRC (2007), of 0.64. Possibly, the higher body fat content in older females in this study contributed to the reduction or dilution of HP per unit of metabolically active tissue (Tedeschi et al. 2002), which led to a decrease in ME_m_ and an increase in *k*_*m*_ The findings of (Pereira et al. 2018) corroborate these results, as they obtained lower *k*_*m*_ (0.58) for Morada Nova sheep slaughtered with an average BW of 28 kg.

NEg is defined as the energy content of the deposited tissue (Garrett et al. 1959), which is a function of the proportion of fat and protein retained in the empty body. Additionally, ruminant feeding Committees also quantify the effect of age, considering maturity in gain requirement models, through the maturity index (NRC 2007; CSIRO 2007), equivalent empty body weight (NASEM 2016), or growth curves (INRA 2018). The findings of these studies show that in the first year of life (1.3 years), the weight gain requirements of e ewes do not change as body weight increases.

From the NE_g_ equation generated in this study, it is estimated, for example, that Santa Ines females gaining 150 g/day and with BW varying from 10 to 40 kg need 0.203 to 0.698 Mcal/day, respectively. If we compare our estimates with the recommendations of the NRC (2007) and INRA () Systems, it becomes evident that their requirement models do not apply to growing Santa Ines females. The NRC (2007) model is based on the degree of maturity to calculate the energy concentration of gain, although the Committee is unclear about how the maturity of the different sexual classes was considered. If the mature weight of Santa Ines females of 32 kg (25% fat in EBW in this study) is considered in the NE_g_ equations of the NRC (2007), it would result in an NE_g_ of 0.856 Mcal/day for females weighing 30 kg and 150 g ADG, a value of 55% higher than that obtained in this study (0.552 Mcal/day). The NEg estimated in this study for a 40 kg ewe with an ADG of 200 g was 0.926 Mcal/day, a value 18% higher than the value reported by Nie et al. (2015) with Dorper ×Hu ewes (0.786 Mcal/day).

On the other hand, INRA (2018) recommends 0.541 Mcal/day for the same range of BW and gain, a value 2% lower than that found in this study. There is great variability between genotypes in the ability to synthesize and store lipids in adipose tissue (Robelin 1986). Furthermore, the maturation rate can be modified by environmental factors (Tedeschi et al. 2004). Therefore, in addition to the different methods of estimating requirements by the Committees (Ma et al. 2022), different types of animals in different environments have variable body composition, which explains the variations in NE_g_ and highlights the difficulty of comparing and using the recommendations of foreign feeding committees.

The variation observed in the parameters of the NE_g_ estimation equation is closely linked to the composition of body weight gain. Females generally exhibit a lower capacity for muscle deposition and reach physiological maturity earlier than males, resulting in an earlier onset of adipose tissue deposition (Deng et al. 2014). Consequently, the energy concentration of gain in females tends to be higher (Lewis and Emmans 2007), which helps explain the higher intercept observed in this study.

The intercept of the equation proposed in this study to estimate the NE_g_ of Santa Ines females was 0.449, which is higher than the values reported by Pereira et al. (2018) for Morada Nova females (0.355), by Pereira et al. (2017) for Santa Ines males (0.203), and by Mendes et al. (2021) for ½ Dorper × ½ Santa Ines males (0.298). Similarly, the EBWG exponent in the present study (1.0284) was higher than those reported in the aforementioned studies (0.990, 0.815, and 0.807, respectively). The exponent in the NE_g_ equation reflects the relationship between energy retention and empty body weight gain (EBWG); a higher exponent indicates a greater energy concentration per unit of gain (Oss et al. 2017), suggesting a lower protein-to-fat ratio in the accreted tissue.

To convert NE_g_ into ME_g_ it is necessary to estimate kg, which represents the efficiency with which ME is used to deposit protein and fat (Commonwealth Scientific and Industrial Research Organisation - CSIRO 2007). In the present study, kg was estimated at 0.40 and was not influenced by age. The kg value found in this study was equal to the value used by the NRC (2007), but is lower than the 0.51 adopted by CSIRO (2007) and the global efficiency value of 0.63 used by INRA (2018).

Normally, a increase in *k*_*g*_ is observed with increasing age (Pereira et al. 2018) when this is obtained by the linear relationship between RE and MEI_g_. In terms of energy efficiency of tissue deposition (Mcal retained/Mcal intake), greater efficiency is expected in older animals due to increasing fat deposition (Rattray and Joyce 1976). The linear relationship between RE and MEIg is limited in conceptual terms when estimating *k*_*g*_ (Tedeschi et al. 2004), as it disregards the different energy densities and deposition efficiencies of protein and fatty tissues (Lindsay et al. 1993), which are grouped according to generally as weight gained.

The adipose tissue is retained more efficiently than protein, and therefore heavier or older animals have a greater energy efficiency of gain (Herbster et al. 2024) This is because the energy value of protein is 5.7 Mcal/kg, but each additional kg of protein is associated with 3.5 to 4.0 times its weight in water CSIRO (2007collab Commonwealth Scientific and Industrial Research Organisation - CSIRO collab, year 2007,), so that the RE in 1 kg of fat-free muscle tissue is only about 1.2 Mcal. Fat, on the other hand, contains 9.4 Mcal/kg, so that the same amount of ME that is deposited in 1 kg of fat is in about 8 kg of muscle (Webster 1977). Furthermore, adipose tissue is characterized by having a slow rate of renewal, while protein tissue has high cell renewal, which makes it an energetically costly tissue with low deposition efficiency (Geay 1984). From the multiple regression of MEI as a function of RE_prot_ and RE_fat_, the values of k_prot_ and k_fat_ were estimated at 0.16 and 0.78, respectively. According to Garrett (1980), partial energy use efficiencies generally range from 0.10 to 0.40 for protein and from 0.60 to 0.80 for fat. Despite the coherence of these results, the values of k_prot_ and k_fat_ are used to a limited extent (Lindsay et al. 1993), as they have little applicability in obtaining energy requirements. Furthermore, the statistical identification of the energetic cost of protein and fat deposition in the same equation is undoubtedly misleading in mechanistic terms (Millward et al. 1976), as the deposition of these tissues is correlated. For example, the efficiency of deposition of a portion of fat inseparable from lean tissue can be counted as RE_prot_, and bias the estimates (Moe 1981).

In addition to the effect of body composition and gain, it is well reported and understood that *k*_g_ also varies depending on feed sources and dietary ME concentration (Garrett 1980). Several nutritional systems consider diet characteristics in *k*_*g*_ estimates (ARC 1980; NRC 2007; CSIRO 2007), although these are less attractive approaches as they disregard the influence of body composition. According to Tedeschi et al. (2010), the combination of information on body composition and dietary energy content may be the best approach for calculating *k*_*g*_. Several efforts have been made to develop mechanistic equations that estimate kg more accurately (Cannas et al. 2006). Tedeschi et al. (2010) combined the following equations: EM (Mcal/day) = RE_prot/_k_prot_ + RE_fat_/k_fat_; RE_prot_ (%) = RE_prot_/RE; and RE_fat_ (Mcal/day) = (1 - RE_prot_) × RE and generated the following model: *k*_*g*_ = (k_prot_ × k_fat_)/[k_prot_ + (%RE_prot_/100) × (k_fat_ − k_prot_)]. Applying the k_prot_ and k_fat_ values obtained in this study to this equation, the following relationship between kg and RE_prot_ (%) is obtained: *k*_*g*_ = 0.12/[0.16 + 0.62 × (% RE_prot_/100)]. From this equation, the *k*_g_ values for Santa Ines females aged 200, 250, and 330 days are calculated as 0.37, 0.37, and 0.39, respectively.

## Conclusions

The energy requirements for maintenance, gain, and efficiency of metabolizable energy utilization for ewes are not altered in the first year of life. The energy requirements for gain are higher than the values recommended by the NRC (2007); however, the energy requirements for maintenance are similar. The models generated in this study are recommended for estimating the energy requirements of Santa Ines hair ewe.

## Data Availability

Data will be made available on reasonable request.
